# Real-time magnetic resonance imaging in pediatric radiology — new approach to movement and moving children

**DOI:** 10.1007/s00247-020-04828-5

**Published:** 2021-02-10

**Authors:** Franz Wolfgang Hirsch, Jens Frahm, Ina Sorge, Christian Roth, Dirk Voit, Daniel Gräfe

**Affiliations:** 1 Department of Pediatric Radiology, University of Leipzig, Liebigstraße 20a 04103 Leipzig, Germany; 2 Biomedizinische NMR, Max-Planck-Institut für Biophysikalische Chemie, Göttingen, Germany

**Keywords:** Children, Fast imaging, Magnetic resonance imaging, Real-time imaging

## Abstract

The recent development of highly undersampled radial gradient echo sequences in combination with nonlinear inverse image reconstruction now allows for MRI examinations in real time. Image acquisition times as short as 20 ms yield MRI videos with rates of up to 50 frames per second with spin density, T1- and T2-type contrast. The addition of an initial 180° inversion pulse achieves accurate T1 mapping within only 4 s. These technical advances promise specific advantages for studies of infants and young children by eliminating the need for sedation or anesthesia. Our preliminary data demonstrate new diagnostic opportunities ranging from dynamic studies of speech and swallowing processes and body movements to a rapid volumetric assessment of brain cerebrospinal fluid spaces in only few seconds. Real-time MRI of the heart and blood flow can be performed without electrocardiogram gating and under free breathing. The present findings support the idea that real-time MRI will complement existing methods by providing long-awaited diagnostic options for patients in early childhood. Major advantages are the avoidance of sedation or anesthesia and the yet unexplored potential to gain insights into arbitrary body functions.

## Introduction

Recent advances in dynamic MRI allow for acquisition times of serial images of 20–30 ms, which correspond to MRI videos with rates of 30–50 frames per second [1]. While preliminary clinical applications suggest new diagnostic opportunities as well as challenges for radiologic practices [2], no studies in the field of pediatric radiology have been reported. This is surprising in view of several specific advantages for MRI examinations in early childhood. In this first overview article we summarize our initial experience gathered since September 2019 in a specialized department for pediatric radiology. To allow the reader to fully appreciate the information of an MRI video even from the printed form of a scientific journal, we have chosen a new format for *Pediatric Radiology* that provides access to video sequences by scanning a QR Code with any personal computer, tablet or smartphone. A first example (Video 1) shows online navigation of the imaging plane by interactive real-time MRI at 30 frames per second, while planning a kidney examination of a 10-year-old boy with urine transport disturbance. Localizer scans serve to freely position the desired cross-section with use of the scanner mouse.

### Video 1


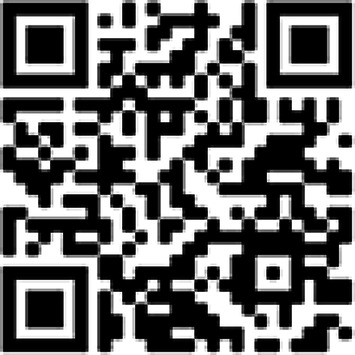


https://pedz.de/rt-supplemental/navigation.mp4

## Description

The study was approved by the local ethics committee. Informed consent was obtained from patients or legal guardians. Patients or legal guardians signed informed consent regarding publishing their data and images.

### Technical prerequisites

According to its definition, real-time MRI combines serial high-speed acquisitions with immediate reconstruction and display of the images with minimum latency. In our implementation this was accomplished by adding a dedicated computer with eight graphical processing units to a standard 3.0-tesla (T) MRI system (Prisma^fit^; Siemens, Erlangen, Germany). The real-time environment is currently only available for Siemens 3-T MR scanners. The measuring protocols appear on the user interface of the scanner like on any other vendor sequence and the resulting image series occur in the usual databank as regular Digital Imaging and Communications in Medicine (DICOM) images.

**Fig. 1:**
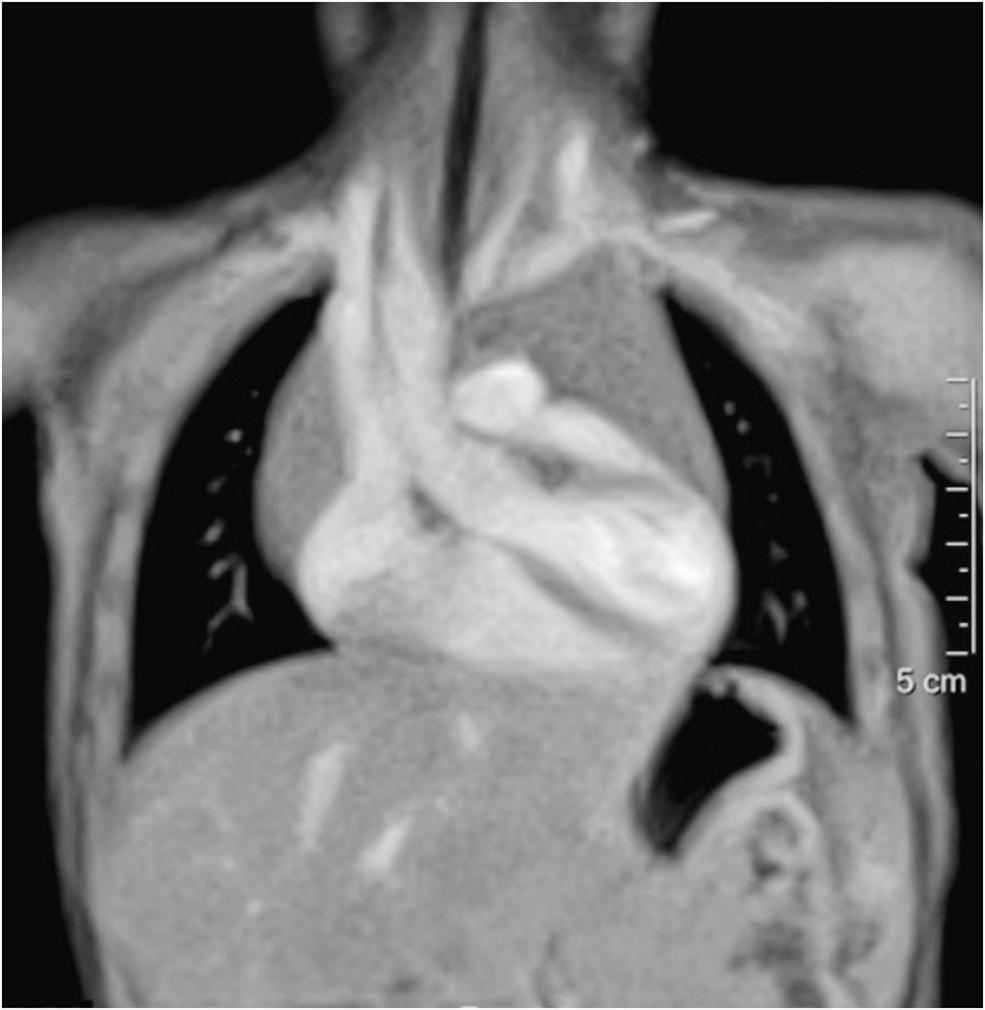
Screenshot taken from a coronal T1-weighted real-time MRI video of the heart in a 5-month-old girl. The examination was performed without electrocardiogram gating and under free breathing. At 30 frames per second (33 ms acquisition time), body movements do not result in image blurring or motion artifacts

The real-time MRI technique used here is based on highly undersampled radial gradient echo sequences in conjunction with a joint reconstruction of images and coil sensitivity maps by nonlinear inversion with temporal regularization to the preceding frame; for details see Uecker et al. [1]. The high temporal fidelity of the method has been experimentally confirmed [3]. Depending on radiofrequency flip angles and gradient schemes, the typical contrast opportunities of gradient echo sequences comprise spin density and T1 contrast as well as steady-state free precession (SSFP) T2-type contrast also known as T2/T1 contrast. Figure 1 shows a selected frame from a T1-weighted real-time MRI study of the heart of a 5-month-old girl. In general, all real-time MRI sequences comply with standard regulations for radiofrequency power deposition and peripheral nerve stimulation. In fact, fast low-angle shot (FLASH)-type acquisitions result in very low specific absorption rate (SAR) values because of their use of small flip angles.

### Suitable real-time magnetic resonance imaging methods for pediatric radiology

The availability of images with very short acquisition times and MRI videos with correspondingly high frame rates can be diagnostically exploited for several examinations and situations. Here we present three approaches to rapid MRI diagnostics in pediatric radiology.

A first method directly focuses on the functional anatomy, or, in other words, on the monitoring of arbitrary, i.e. also aperiodic, dynamic processes. For example, this holds true for studies of the oral cavity during speaking [4] or swallowing [5, 6] and also applies to joint movements [7]. Moreover, in contrast to conventional electrocardiogram (ECG)-gated cine techniques, which retrospectively merge data from multiple heartbeats into a single cardiac cycle, myocardial movements can now be recorded in real time. These applications have been extended to real-time phase-contrast flow MRI [8, 9]. Multi-slice real-time MRI videos can be obtained in a sequential manner.

A second method refers to quantitative tissue characterization, more precisely to rapid T1 mapping. This is accomplished by a brief FLASH-type real-time MRI acquisition of typically 4 s, preceded by a 180° inversion pulse. The resulting image series defines a single-shot inversion-recovery dataset that has been shown to yield highly accurate T1 maps by pixel-wise fitting [10, 11]. When using slice-selective inversion pulses, this method can be extended to multi-slice T1 mapping of whole organs (i.e. 4 s per slice).

A third method allows for rapid scanning of a large volume by advancing the slice position of each frame of a cross-sectional real-time MRI study by a certain percentage of the slice thickness [12]. The resulting spatial overlap generates sufficient similarity of successive, i.e. neighboring, sections and therefore allows for the same nonlinear inverse reconstruction as for dynamic real-time MRI. Because of the short acquisition times, individual frames are robust against motion, which is in contrast to conventional 3-D MRI techniques. Typically, volume coverage of a child’s brain with 200–300 overlapping images can be performed in a few seconds per orientation.

### Body movements

Until now, it has not been possible to dynamically evaluate aperiodic body movements such as, for example, the mobility of the cervical spine. Video 2 depicts a sagittal T1-weighted real-time MRI recording of a 12-year-old boy with paresthesia in the right arm and suspected vertebral gliding; the video demonstrates a continuous motion without constriction of the craniocervical junction. The movie further reveals the pulsatile nature of the cerebrospinal fluid (CSF) dynamics, which is important in a variety of pathological conditions including Chiari malformation.

#### Video 2


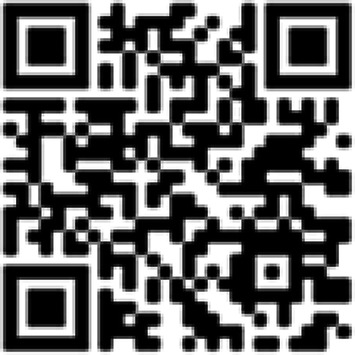


https://pedz.de/rt-supplemental/spine.mp4

Similarly, conventional MRI techniques cannot visualize post-traumatic joint constellations, which for children means they eventually require X-ray fluoroscopy. In contrast, Video 3 shows a coronal T1-weighted real-time MRI recording of a 3-year-old girl without sedation. The examination served as a postoperative control after repositioning of the left hip and the video easily documents correct position and movement of the femoral head. Abduction and adduction of the leg is performed by a person lying in prone position on the patient table.

#### Video 3


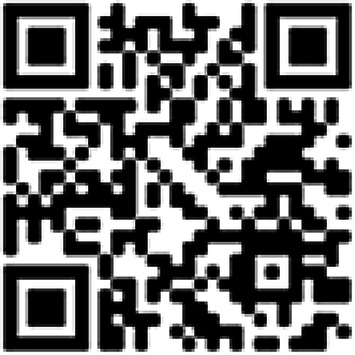


https://pedz.de/rt-supplemental/hip.mp4

### Speaking, swallowing and beyond

Monitoring the complex motility of tongue, velum and soft palate during speaking is critically important in logopedics as well as for pre-surgical planning and post-surgical evaluation of speech problems. The case shown in Video 4 refers to a child who learned to correctly vocalize words (e.g., “krokodil,” the German equivalent of “crocodile”) after surgical fixation of the velum to the posterior wall of the oral cavity.

#### Video 4


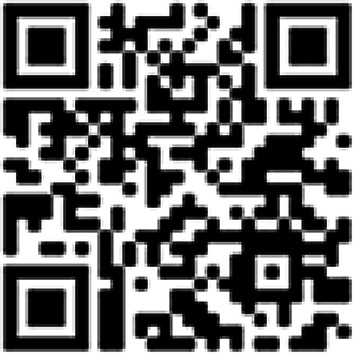


https://pedz.de/rt-supplemental/crocodile.mp4

Swallowing processes can be studied by voluntary swallows of pineapple juice because it contains an inherent concentration of paramagnetic manganese, which serves as an MRI contrast agent for T1-weighted images. This is demonstrated in Video 5 in a healthy subject. However, this diagnostic can play a role in all coordination disorders of the swallowing process for which fluoroscopy has been used, as well as in the diagnosis of gastroesophageal reflux [13]. Children after esophageal atresia should also benefit from MRI swallowing studies.

#### Video 5


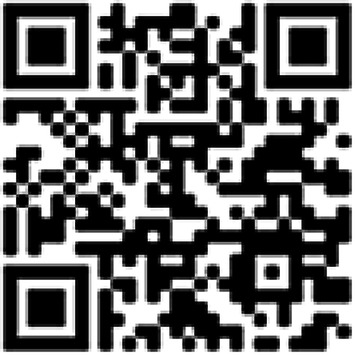


https://pedz.de/rt-supplemental/swallow.mp4

Evaluation of the filling of the stomach and passage of the fluid into the small bowel is another diagnostic option, analogous to X-ray fluoroscopy but without ionizing radiation and access to 3-D information. The case shown in Video 6 represents a coronal T1-weighted volume coverage scan of a 6-week-old boy with suspected anastomotic stricture after duodenojejunostomy in duodenal atresia, obtained within 11 s without sedation.

#### Video 6


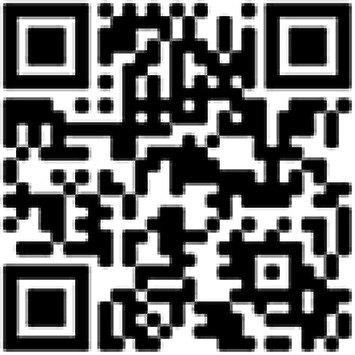


https://pedz.de/rt-supplemental/duodenum.mp4

### Cardiac magnetic resonance imaging

Cardiac MRI studies in real time appear as a most natural and obvious application in the field of pediatrics. Such measurements do not require ECG or self-gating techniques and can be performed during free breathing without the use of navigator scans. Moreover, when based on FLASH-type T1-weighted versions at 3.0 T, the short gradient-echo times do not require time-consuming shimming procedures, while completely avoiding SSFP-inherent banding artifacts. Preliminary applications in adults have focused on those with arrhythmias [14], where ECG synchronization is barely possible and commonly leads to poor image quality. Here, Video 7 illustrates the case of a 5-day-old boy with interrupted aortic arch where the slice position is automatically shifted every 3 s to cover a larger volume in sequential recordings. Despite pronounced movements of the child, the wall of the thorax and abdomen appear without blurring. The second example in Video 8 is an 8-month-old boy with right aortic arch and aberrant left subclavian artery leading to alterations of the distal trachea and right main bronchus, while the case in Video 8 refers to a 17-year-old with funnel chest. The application of a vacuum bell at increasingly negative pressure demonstrates the elevation of the sternum with decompression of the heart.

#### Video 7


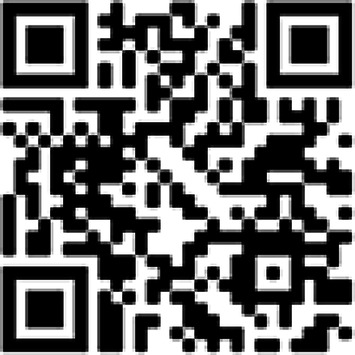


https://pedz.de/rt-supplemental/iaa.mp4

#### Video 8


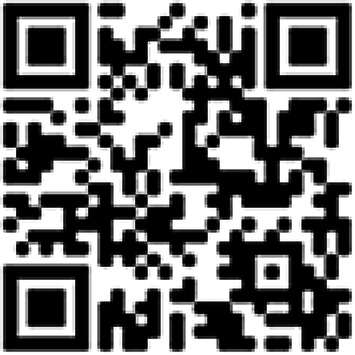


https://pedz.de/rt-supplemental/lusoria.mp4

### Phase-contrast flow magnetic resonance imaging

**Fig. 2:**
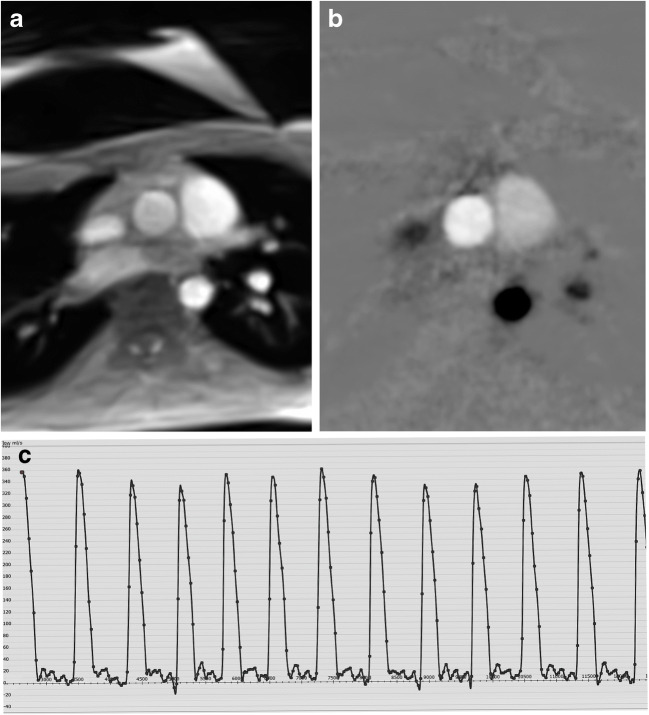
Real-time phase-contrast flow MRI of the ascending aorta in a 17-year-old male adolescent with funnel chest and vacuum bell at low pressure (same child as in Online Supplementary Material 9). **a** Anatomical image. **b** Velocity map. **c** Time course of evaluated flow volume. Please note the reduced phase noise in (**b**) from the use of a model-based reconstruction technique and the respiration-dependent variation of the flow volume in (**c**)

Access to blood flow in real time is a logical extension of anatomical real-time MRI and requires the adoption of phase-contrast flow MRI principles to determine velocity maps for through-plane flow [8]. Extending conventional phase-contrast flow MRI, the use of a model-based reconstruction eliminates the “salt-and-pepper” noise in regions such as the lung that are without MRI signal support, and it further improves the spatio-temporal resolution [9]. Figure 2 presents the results of a real-time flow MRI study of the ascending aorta in the same child as in Video 9. The true time courses of flow volume over multiple heartbeats reveal respiration-dependent variations. Clinically relevant evaluations in pediatric cardiology are the determination of flow volumes (or rates) of the large thoracic vessels in people with arrhythmias as well as in young anxious children.

#### Video 9


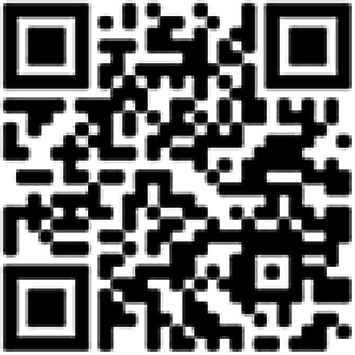


https://pedz.de/rt-supplemental/funnel.mp4

In addition to blood flow measurements at high velocities, real-time flow MRI techniques provide access to the low-velocity dynamics of the CSF. Such studies in healthy adults recently demonstrated respiration to be a major regulator of spinal CSF flow mildly modulated by cardiac pulsatility [15].

### Rapid T1 mapping

**Fig. 3:**
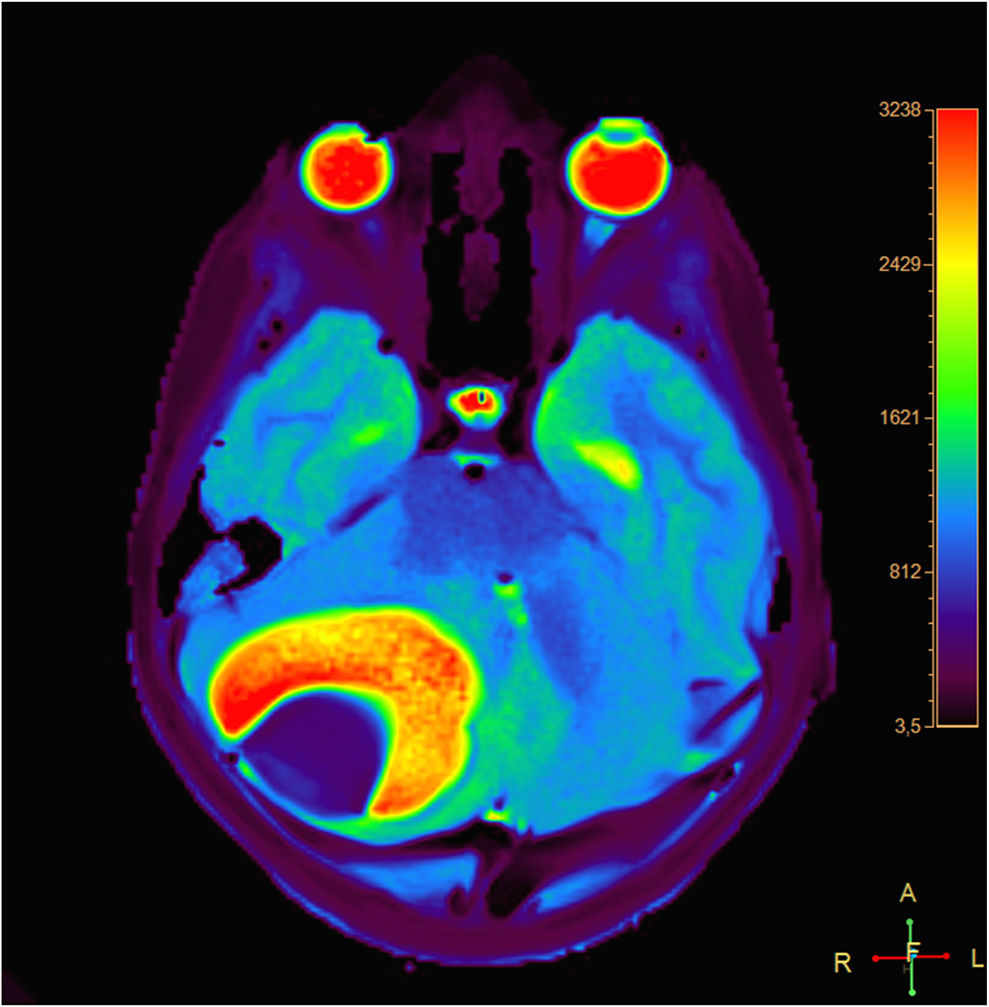
Transverse quantitative T1 map of a 6-year-old boy with pilocytic astrocytoma in the right cerebellar hemisphere. High T1 values (2,800 ms) in cystic tumor compartments are coded in red, and very low values (480 ms) in solid tissue are coded in deep blue

The ability to obtain quantitative maps of absolute T1 relaxation times in arbitrary tissues within only 4 s per map emerges as an attractive tool that might serve as a biomarker. Such studies are possible on all parenchymatous organs and can contribute to tumor characterization in the context of multiparametric measurements. For example, T1 maps of age-dependent differences in white matter during infancy and childhood can be used to assess delayed myelination in an objective manner. Figure 3 presents a color-coded T1 map of a 6-year-old boy with pilocytic astrocytoma, where high T1 values (red=2,800 ms) and low T1 values (blue=480 ms) distinguish cystic tumor compartments from solid tissues. The use of a slice-selective inversion pulse extends the measurement to an automatic multi-slice acquisition covering the entire brain. Video 10 depicts such a series of 35 contiguous T1 maps (4 s each) of the brain of a 5-year-old girl with Neurofibromatosis Type 1, revealing multiple hamartomas, especially within the left globus pallidus. The total scan time of 2 min 20 s was much shorter than achievable with conventional methods.

#### Video 10


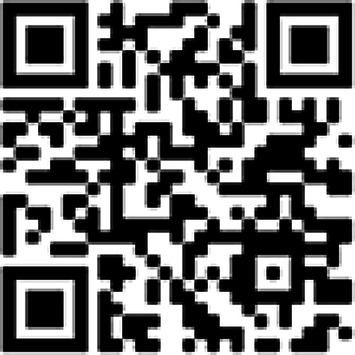


https://pedz.de/rt-supplemental/t1map

### Rapid volume coverage

**Fig. 4:**
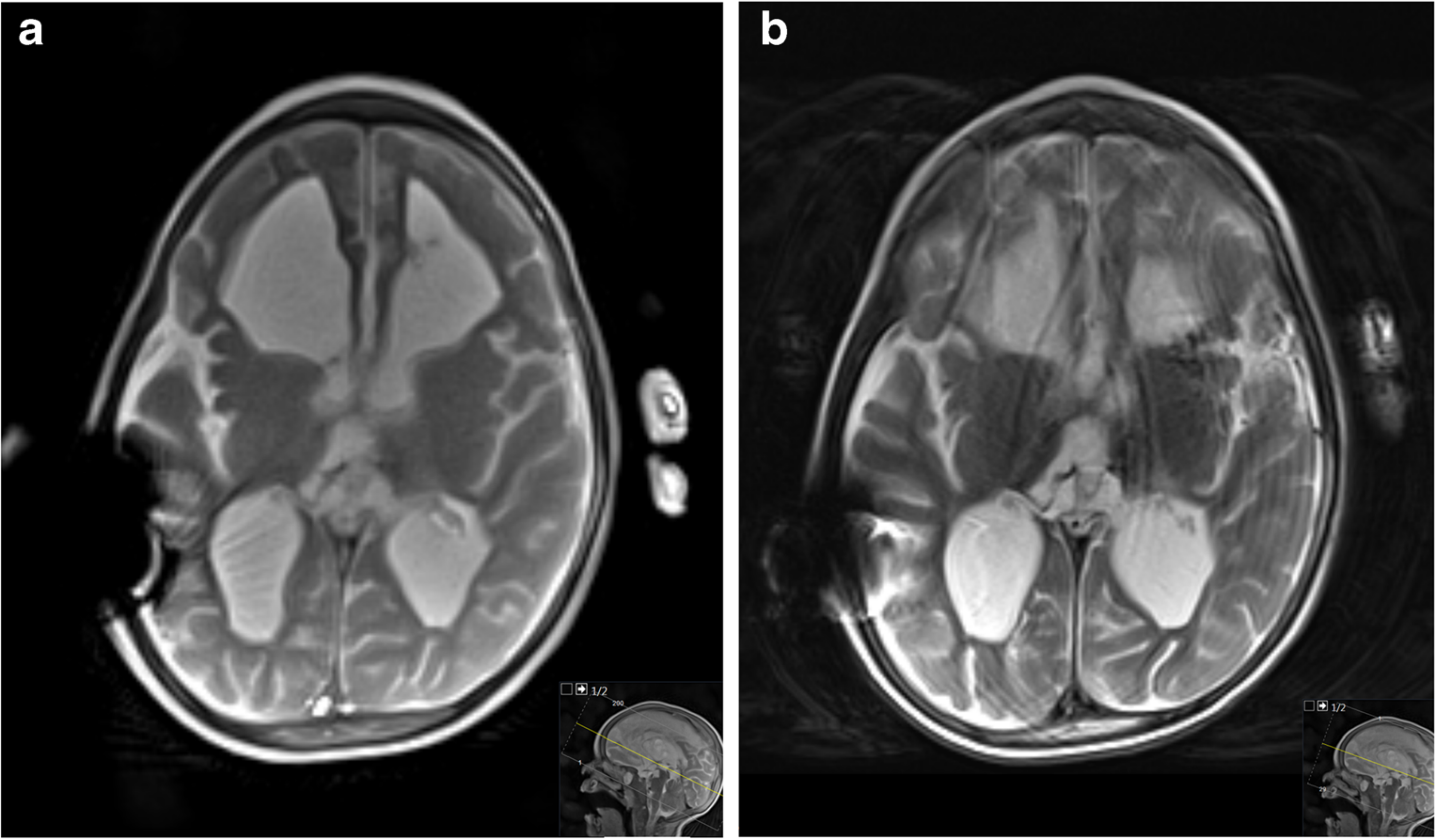
Intracranial bleeding and a ventriculoperitoneal shunt causing local susceptibility-induced signal void in a 2.3-year-old boy. **a, b** Comparison of (**a**) a transverse T2/T1-weighted volume coverage scan (11 s) based on cross-sectional real-time MRI without motion artifacts and (**b**) a conventional T2-weighted fast spin-echo (FSE) sequence (38 s) with motion artifacts

In pediatric radiology the need for volumetric assessments, for example by 3-D MRI with measuring times of minutes, is often compromised by involuntary movements or requires the use of sedation or anesthesia. The new volume coverage scan sequences by real-time MRI with automatic slice advancement overcome this problem: the method is not only motion-robust because individual frames are acquired within tens of milliseconds, but it is also fast in scanning large fields-of-view with overlapping images [12]. Figure 4 demonstrates the reduced sensitivity to motion in a 2.3-year-old boy after intracranial bleeding and ventriculo-peritoneal shunt by comparing a selected frame from a T2/T1-weighted volume coverage scan with a conventional T2-weighted fast spin-echo image. A full evaluation of the brain in all three orientations is shown in Video 11 for the case of a 4-year-old girl with Blake pouch cyst. Each of the transverse, coronal and sagittal T2/T1-weighted volume coverage scans with up to 300 overlapping images (3-mm section thickness, 0.5-mm slice shift) were obtained within a total scan time of 7–15 s. Accordingly, the overall measurement time of the patient including planning could be reduced to less than a minute.

#### Video 11


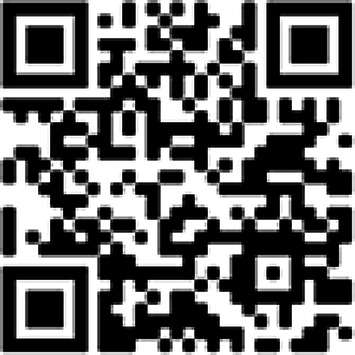


https://pedz.de/rt-supplemental/vc_3planes.mp4

The rapid volume coverage scans have demonstrated good to very good image quality in the evaluation of inner and outer CSF spaces in the brain (Gräfe et al. 2020, article in press in Pediatric Radiology). In all cases the diagnostic question could be answered. Moreover, in comparison to CT and US, the method offers more comprehensive information, so that it is now part of our routine portfolio. It should especially be noted that for all children up to 6 years of age, the MRI examination could be performed without sedation when a parent or another person mildly fixated the child with the hands. Finally, a correspondingly adjusted version might in the future be applied to fetal imaging and complement or even replace conventional T2-weighted sequences for a fretful and moving fetus, as already supported by preliminary trials.

## Discussion

To the best of our knowledge this is the first report about the use of real-time MRI in pediatric radiology. This is surprising in view of its several advantages such as extremely short examinations, insensitivity to motion, very low energy deposition for FLASH-type contrast, and avoidance of sedation in children up to 6 years of age. As demonstrated in this initial overview, the technology offers a variety of applications, all of which were met by clinical indication. Our preliminary experience in these cases clearly supports the idea that real-time MRI without sedation or anesthesia could gain significant importance for MRI studies in early childhood. However, because of some compromises in spatial resolution and contrast, real-time MRI applications are likely to complement rather than replace conventional state-of-the-art MRI techniques in older children. Nevertheless, this first report identifies several clinical indications where real-time MRI offers more rapid and better information, in particular when compared to US and its well-known limitations. Similar arguments hold true for CT because of the exposure to ionizing radiation and the reduced soft-tissue contrast. As a consequence, there might be a change of paradigm in pediatric radiology: if a brief MRI examination yields sufficient information to allow for a conclusive diagnosis, then the role of MRI in general and the access to suitable MRI techniques need to be reconsidered, at least for children up to the age of 6 years. Of course, this process requires extensive scientific study to fully understand all of the technical and clinical pros and cons of the novel real-time MRI technology and its dependent applications. Such study, we hope, would substantiate and confirm our current expectations as a prerequisite for routine clinical use. The present findings support the idea that real-time MRI will complement existing methods by long-awaited diagnostic options for patients in early childhood. Major advantages are the avoidance of sedation or anesthesia and the unexplored potential to gain insights into arbitrary body functions.
